# Addressing the relationship between cardiac hypertrophy and ischaemic stroke: an observational study

**DOI:** 10.1186/1755-7682-5-32

**Published:** 2012-12-15

**Authors:** Chaturaka Rodrigo, Sajitha Weerasinghe, Vijayabala Jeevagan, Senaka Rajapakse, Godwin Constantine

**Affiliations:** 1 Department of Clinical Medicine, Faculty of Medicine, University of Colombo, Colombo, Sri Lanka; 2 National Hospital of Sri Lanka, Colombo, Sri Lanka

## Abstract

**Introduction:**

Research over the last decade has recognized left ventricular hypertrophy as a risk factor for major cardiovascular events including stroke. While cardiac magnetic resonance imaging is the best modality to quantify left ventricular hypertrophy, echocardiographic calculation of left ventricular mass index is a reasonable alternative.

**Methods:**

We carried out a hospital based prospective study to identify the prevalence of left ventricular hypertrophy, assessed using echocardiography, in patients presenting with ischaemic strokes. This is the first study that attempted to quantify this issue in a cohort of Sri Lankan patients. The study was carried out in the National Hospital of Sri Lanka over a period of 6 months.

**Results:**

A total of 55 patients (44 males, 80%) (mean age: 62.3, range: 48–82 years) with ischaemic strokes were studied. Of them, only 38 could be mobilized to measure the height and weight to calculate the left ventricular mass index. Of the rest, only one person had the electrocardiographic criteria for left ventricular hypertrophy. Of the 38 patients evaluated, 29 (76.3%) had left ventricular hypertrophy while 19 (50%) had severe hypertrophy.

**Discussion and conclusions:**

The rates of left ventricular hypertrophy reported in similar studies in other countries vary between 25-62%. Given the high prevalence of left ventricular hypertrophy reported in this study and its recognition as a risk factor for stroke recently, together with the availability of effective treatment for risk reduction, the cost effectiveness of population screening should be evaluated. Further studies are planned in this regard.

## Introduction

Left ventricular hypertrophy (LVH) is associated with cardiovascular morbidity and mortality [1]. Similarly, therapies that cause regression of LVH result in a reduction of cardiovascular events and related mortality [2]. Several studies have demonstrated LVH to be a risk factor for ischaemic stroke that is similar to the more traditional risk factors such as sex, age, diabetes and hypertension [3,4]. The exact mechanism of how LVH leads to increased risk of stroke is yet unclear, although flow and functional disturbances created by altered ventricular geometry may play a role. We performed an observational study to determine the presence of LVH in patients presenting with ischaemic strokes in Sri Lanka. Although LVH has been established as a potential risk factor for ischaemic strokes, there may be regional and racial differences that may modulate its impact by means of genetics and life style. The prevalence of LVH among Sri Lankan patients with ischaemic strokes has not been assessed before.

## Methods

We assessed all patients presenting with ischaemic stroke to two medical units of the National Hospital of Sri Lanka (NHSL) with echocardiography over a 6 month period. NHSL is the premier tertiary care referral center of the country. The patients were recruited by the investigators after informed voluntary consent, and their demographic, anthropometric and health related data were extracted in to a data sheet. Ischaemic strokes were diagnosed by history, examination and by exclusion of a haemorrhage with computed tomography (CT) scan of the brain. All patients were clinically categorized according to the Oxfordshire Community Stroke Project classification [5]. All eight medical units of NHSL receive patients with stroke on rotation, hence there was no selection bias in patient recruitment. The standard treatment protocols were followed in treating the patients; the investigators did not intervene in their care. All patients who consented to the study underwent electrocardiographic recording and echocardiography within 2–6 days of their hospital admission. LVH was defined electrocardiographically according to Cornell criteria [6]. The echocardiographic analyses were performed by a single cardiologist for the entire study using the same machine (GE Vivid 3 with a 3.5 MHz transducer) according to the recommendations by American Society of Echocardiography. Data were collected regarding cardiac valves, chamber sizes, E/A ratio, wall thickness, motion abnormalities and relaxation times. Left ventricular mass (LVM), left ventricular mass index (LVMI) and relative wall thickness (RWT) were calculated using anthropometric data, left ventricular end diastolic diameter, posterior wall thickness and interventricular septal thickness (at end diastole) [7,8]. The previously established cutoffs were used to define normal and abnormal LVMI [7,8]. Though LVMI is considered a better option than left ventricular wall thickness to assess LVH, its calculation requires the height and weight data of patients [1]. Unfortunately some patients were too ill to be moved out of bed to record these parameters accurately, which limited the number of patients included in the final analysis. Ethical clearance for the study was obtained from the Ethics Review Committee of the Faculty of Medicine, University of Colombo.

## Results

A total of 55 patients (44 males, 80%) (mean age; 62.3, range; 48–82 years) with ischaemic strokes were recruited in to the study during the six months. Their demographic and clinical profile is summarized in Table 1. Clinically, most infarcts were categorized as lacunar infarcts. Only 13 (23.6%) patients had a visible infarct on the non-contrast CT scan that was performed to exclude an intracranial haemorrhage.

**Table 1:** Demographic and clinical profile of patients with ischaemic strokes recruited to the study

**Variable**	**Number**	**Percentage (%)**
**Gender**		
Male	44	80
Female	11	20
**Risk factors**		
Diabetes	9	16.4
Hypertension	32	58.2
Hypercholestrolaemia	7	12.7
Stroke	7	12.7
Transient ischaemic attack	3	5.5
Atrial fibrillation	0	0
Heart failure	0	0
Ischaemic heart disease	9	16.4
Smoking	17	30.9
No identifiable risk factors	13	23.6
Clinical classification of strokes*		
TACI	0	0
PACI	20	36.4
POCI	8	14.5
Lacunar infarct	27	49.1

*TACI; total anterior circulation infarct, *PACI*; partial anterior circulation infarct, *POCI*; posterior circulation infarct.

Regarding the cardiovascular assessment, the electrocardiographic tracings fulfilled the voltage criteria for left ventricular hypertrophy in only 8 patients. However when assessed with echocardiography, the numbers fulfilling criteria for LVH were much higher. Of the 55 patients, 38 could be mobilized to measure the height and weight to calculate the LVMI, LVM and RWT. Of the rest, one person had the electrocardiographic criteria for LVH. The findings are summarized in Table 2. The remaining 16 could not be assessed accurately for LVH by a reliable test. Overall it can be stated that at least 30 (54.5%) of the sample had LVH and of the total subset that had their ventricular parameters reliably assessed by calculation of LVMI (n = 38), 29 (76.3%) had an abnormal reading. Severe LVH was seen in 19 (50%) of them.

**Table 2:** Echocardiographic evidence of left ventricular hypertrophy in the sample

**Parameter**	**Number (n- 38)**	**Percentage (%)**
LVMI*		
No hypertrophy	8	21.1
Mild hypertrophy	6	15.8
Moderate hypertrophy	4	10.5
Severe hypertrophy	19	50.0
RWT**		
Concentric hypertrophy	25	65.8
Eccentric hypertrophy	4	10.5
Concentric remodeling	7	18.4
Normal	1	2.6

*LVMI (g/m^2^); for males: normal (49–115), mildly hypertrophic (116–131), moderately hypertrophic (132–148), severely hypertrophic (>149); for females: normal (43–95), mildly hypertrophic (96–108), moderately hypertrophic (109–121), severely hypertrophic (>121).

**RWT allows further classification of LV mass increase as either concentric hypertrophy (RWT >0.42) or eccentric hypertrophy (RWT ≤0.42).

## Discussion

The importance of LVH as a risk factor for stroke has been highlighted in many studies in both developing and developed countries. The rates of LVH reported in these studies in stroke patients vary between 25-62% [4,9-11]. The evidence from Israeli ischaemic heart diseases project which followed up nearly 10,000 people over a 21 year period (a follow up of nearly 10,000 person years related to ischaemic stroke) established that LVH had an increased risk for ischaemic stroke (hazard ratio; 2.15) [12]. In the Jackson cohort of the Atherosclerotic Risk in Communities (ARIC) study in USA, LVH was independently associated with developing an ischaemic stroke (after adjusting for age, sex, hypertension, smoking, diabetes, body mass index and HDL cholesterol levels) [11]. The adjusted hazard ratio for stroke per 10 g/m^2^ increment in LVMI was 1.15 [95% confidence interval (CI); 1.02 to 1.28]. In another community based follow up of 2313 middle-aged men in Sweden, 421 developed a stroke or a transient ischaemic attack (TIA) over a follow up of 32 years. Echocardiographically demonstrated LVH and smoking were associated risk factors for either outcome [13]. The Dutch TIA trial study group also established that electrocardiographic evidence of LVH is an independent risk factor for TIAs [14].

Of hospital based studies, Di Tullio and colleagues, in a case control study of 394 patients with first episode ischaemic stroke (and 413 race, age and sex matched controls), showed that echocardiographically determined LV mass was a significant risk factor for strokes [15]. LV relative wall thickness was also independently associated with stroke after correcting for LV mass. Concentric hypertrophy had the greatest risk [adjusted odds ratio (OR), 3.5; 95% confidence interval [CI], 2.0 to 6.2 followed by eccentric hypertrophy (adjusted OR, 2.4; 95% CI, 2.0 to 4.3). Castilla-Guerra and colleagues assessed 203 consecutive patients with acute ischaemic strokes or transient ischaemic attacks and found that 42.3% of the sample had LVH [9]. In a study by Amin et al. 25% of the sample of 120 patients with ischaemic stroke were found to have LVH. Stroke was confirmed clinically as well as by magnetic resonance imaging. LVH was more common in iscahemic stroke than in subarachnoid haemorrhage (17%) and intracranial haemorrhages (22%) [10]. A case control study in Manhattan, New York, showed that while having a high left ventricular mass (LVM) was significantly increased the risk of ischaemic stroke, being physically active contributed to a risk reduction that was more marked in patients with increased LVM than those with normal LVM [16].

Of the studies from developing world, Khan and colleagues recorded a 3.6% prevalence of LVH among a series of 55 patients with ischaemic stroke in Pakistan [17]. A case control study in Brazil on first episode ischaemic stroke patients showed that those with LVH had an odds ratio of 20.3 (95% CI; 8.8-46.4) favouring stroke. Another case control study In Taiwan of 238 hospitalized patients with first ever ischaemic stroke showed that electrocardiographically determined LVH was a significant risk factor for stroke [odds ratio (OR): 3.9, 95% CI; 2.02 to 7.39] [18]. Another study from Taiwan of 228 patients with first episode ischaemic stroke and a similar number of healthy age and sex matched controls confirmed the increased risk of LVH for stroke (OR: 2.7, 95% CI = 1.18-6.16) [19]. The validity of using electrocardiographic criteria (Cornell and Sokolow-Lyon) for assessing LVH in stroke patients has been established by Kohsaka and colleagues who showed that in their study of 177 patients with first episode ischaemic stroke, ECG determined LVH was a risk factor with an odds ratio of 2.06, (95% CI, 1.26-3.35) assessed by Cornell criteria and 2.12, 95% CI 1.05-4.30 by Sokolow-Lyon criteria [20]. In our study the percentage of stroke patients with an above reference range LVMI was as high as 76.3% and most of them (65.8%) had concentric hypertrophy. This is the first study to record LVMI in strokes patients in Sri Lanka. The sensitivity of electrocardiography was low compared to echocardiography in diagnosing LVH.

How LVH predisposes to stroke and other cardiovascular events is still under speculation but multiple mechanisms are proposed. LVH increases the work load of the ventricle and the wall tension by alteration of ventricular geometrics [1]. This is further complicated by increasing myocardial fibrosis that contributes to diastolic dysfunction limiting end diastolic filling capacity and increasing left ventricular end diastolic pressure. These sequences of events are controlled by non-modifiable factors such as genetics and modifiable factors such the renin, angiotensin, aldosterone axis. The increased work load of the ventricles also increases the risk of vascular insufficiency to the inner parts of the hypertrophied myocardium leading to areas of myocardial ischaemia or infarcts. These may create small areas of dyskinetic or hypokinetic myocardium which may serve as areas of origin for small thrombi. However, not all patients have symptoms and some may be undiagnosed till a major event happens. In our study, out of 23 patients who did not have a prior diagnosis of hypertension, 17 (74%) had LVH at the time they were admitted with a stroke.

The role of genetics in increasing vulnerability to hypertension, LVH and ischaemic stroke has been studied in several recent studies. Lanni and colleagues have demonstrated that in hypertensive patients, polymorphism of the platelet glycoprotein IIIa gene (GPIIIa Pl^A2^) increases the risk of ischaemic stroke in high risk groups (with three or more risk factors) [21]. LVH is often a consequence of long standing hypertension. On the same topic, a genome-wide analysis published in 2007 showed a single nucleotide polymorphism of the calcium/calmodulin dependent kinase IV (CaMK IV) gene to have an association with hypertension [22]. Santulli and colleagues conducted an animal study on mice with double deletion of CaMK IV and showed them to have larger hearts and concentric hypertrophy due to septal and posterior wall thickness compared to mice without the deletion [23]. The CaMK IV deleted mice were also more susceptible to effects of chronic ischaemia leaving them vulnerable to myocardial infarction and possibly stroke. This was probably mediated through the effect of CaMK IV on endothelial nitric oxide synthase activity. The possible molecular mechanisms responsible for the development of LVH in hypertension have been a focus of studies in the last few years. The role of nuclear factor kappa B (NF-κB) has emerged as a focal point in this regard. NF-κB is a transcription factor involved in multiple cellular functions ranging from cell division, apoptosis to immune functions. It is shown to influence the hypertrophic growth of cardiac myocytes in response to stimuli such as endothelin I, norepinephrine and angiotensin II [24]. It has been further demonstrated in animal studies that inhibition of NF-κB signaling leads to a reduction of LVH in hypertensive rats and prevents the development of cardiac hypertrophy in normotensive rats [25]. These findings may have an implication in delineating the molecular and genetic mechanisms of LVH and designing of preventive measures for its complications such as strokes.

This brings forth the issue of community screening for LVH. In theory it appears to be a good option as unidentified LVH is a risk factor for major cardiovascular events as well as other more subtle manifestations such as cognitive impairment which can have a significant cumulative effect on quality of life [26]. In addition, there are effective pharmacological means of treating LVH with angiotensin receptor blockers (ARB) and angiotensin converting enzyme inhibitors (ACEI) [1]. Regression of LVH with treatment is associated with reduced risk of major cardiovascular events including stroke [27]. However, the mode of screening for LVH is an issue. Electrocardiography has high specificity but low sensitivity. Echocardiography is a better option but has problems of availability and cost concerns. Cardiac MRI is the best choice but is prohibitively costly for a screening tool. It is our opinion that cost effectiveness of primary prevention by early identification of LVH by echocardiography must be evaluated against the treatment costs and rehabilitation costs of patients suffering major cardiovascular accidents. As it may be impossible to screen all, risk stratification scoring systems can be developed to identify high risk individuals to screen (based on medical history and symptoms).

This was a preliminary study to identify the prevalence of LVH in patients presenting with ischaemic strokes to hospital. As the results showed a significantly high percentage of LVH, further evaluation of the problem is planned in two stages: a) a case control study to identify the impact of LVH on cerebrovascular accidents after controlling for confounding factors, and b) a community based prospective cohort study on screening for risk factors for stroke (including LVH) and comparison of event rates over time. Such an approach will provide concrete evidence on the role and cost effectiveness of echocardiography as a screening tool to identify LVH in the community.

## Limitations

Our study does not provide data to justify recommendation of community screening for asymptomatic people as it was a hospital based study that did not have a control population for comparison. Therefore we plan to conduct further studies to resolve this issue as mentioned above. The numbers were limited to carry out a factorial analysis of different subgroups. The effect of risk factors such as diabetes, hypertension and hypercholesterolaemia are better analyzed against dependent variables such as LVMI by taking the actual values of HbA1c, mean blood pressure and cholesterol level (rather than restricting to a dichotomous definition of presence or absence of a particular risk factor) [28-31]. Since we did not follow up these patients we could not carry out such an analysis in this study.

## Conclusions

This hospital based study of patients with ischaemic stroke showed a high percentage (76%) of left ventricular hypertrophy (as confirmed by echocardiographic calculation of left ventricular mass index) in the sample. Fifty percent had severe LVH. The importance of LVH as an independent risk factor for stroke has been shown by similar studies in other countries but this is the first study to document this in Sri Lanka. Given the fact that a significant proportion of patients with LVH are asymptomatic and treatment of LVH reduces the risk of cardiovascular events, the cost effectiveness of population screening with echocardiography needs to be evaluated.

## Competing interests

The authors declare that they have no competing interests.

## Authors’ contributions

GC conceptualized the study. CR, SW, SR and GC planned the study. VJ, CR and SW did the data collection and patient recruitment. GC did the echocardiographic analysis. CR wrote the first draft. All authors reviewed the final manuscript. All authors read and approved the final manuscript.

## Authors’ information

SW and VJ are registrars attached to the University Medical Unit, National Hospital of Sri Lanka. CR is lecturer in Medicine and SR is Professor in Medicine of the Department of Clinical Medicine, Faculty of Medicine, University of Colombo, Sri Lanka. GC is Senior lecturer and Consultant Cardiologist attached to the same department.
